# *In Vivo* Bioluminescence Imaging for Prolonged Survival of Transplanted Human Neural Stem Cells Using 3D Biocompatible Scaffold in Corticectomized Rat Model

**DOI:** 10.1371/journal.pone.0105129

**Published:** 2014-09-08

**Authors:** Do Won Hwang, Yeona Jin, Do Hun Lee, Han Young Kim, Han Na Cho, Hye Jin Chung, Yunwoong Park, Hyewon Youn, Seung Jin Lee, Hong J. Lee, Seung U. Kim, Kyu-Chang Wang, Dong Soo Lee

**Affiliations:** 1 Department of Nuclear Medicine, Seoul National University College of Medicine, Seoul, Korea; 2 Department of Molecular Medicine and Biopharmaceutical Science, WCU Graduate School of Convergence Science and Technology, Seoul National University, Seoul, Korea; 3 University of Miami School of Medicine, Miami Project to Cure Paralysis, Department of Neurological Surgery, Miami, Florida, United States of America; 4 College of Pharmacy, Ewha Womans University, Seoul, Korea; 5 Cancer Imaging Center, Seoul National University Cancer Hospital, Seoul, Korea; 6 Cancer Research Institute, Seoul National University College of Medicine, Seoul, Korea; 7 Medical Research Institute, Chung-Ang University College of Medicine, Seoul, Korea; 8 Division of Pediatric Neurosurgery, Seoul National University Children's Hospital, Seoul, Korea; Stanford University School of Medicine, United States of America

## Abstract

Stem cell-based treatment of traumatic brain injury has been limited in its capacity to bring about complete functional recovery, because of the poor survival rate of the implanted stem cells. It is known that biocompatible biomaterials play a critical role in enhancing survival and proliferation of transplanted stem cells via provision of mechanical support. In this study, we noninvasively monitored *in vivo* behavior of implanted neural stem cells embedded within poly-l-lactic acid (PLLA) scaffold, and showed that they survived over prolonged periods in corticectomized rat model. Corticectomized rat models were established by motor-cortex ablation of the rat. F3 cells expressing enhanced firefly luciferase (F3-effLuc) were established through retroviral infection. The F3-effLuc within PLLA was monitored using IVIS-100 imaging system 7 days after corticectomized surgery. F3-effLuc within PLLA robustly adhered, and gradually increased luciferase signals of F3-effLuc within PLLA were detected in a day dependent manner. The implantation of F3-effLuc cells/PLLA complex into corticectomized rats showed longer-lasting luciferase activity than F3-effLuc cells alone. The bioluminescence signals from the PLLA-encapsulated cells were maintained for 14 days, compared with 8 days for the non-encapsulated cells. Immunostaining results revealed expression of the early neuronal marker, Tuj-1, in PLLA-F3-effLuc cells in the motor-cortex-ablated area. We observed noninvasively that the mechanical support by PLLA scaffold increased the survival of implanted neural stem cells in the corticectomized rat. The image-guided approach easily proved that scaffolds could provide supportive effect to implanted cells, increasing their viability in terms of enhancing therapeutic efficacy of stem-cell therapy.

## Introduction

Traumatic brain injury (TBI), often defined as an acquired brain injury or simply a brain injury, is the leading cause of mortality and disability among young adults and elderly people, and it occurs when the brain is damaged by a sudden trauma such as those associated with falls, motor vehicle accidents, and surgical operations for epilepsy treatment [1], [2]. Treatment of TBI has been largely dependent on use of various types of neuronal progenitors, or stem cells, to restore the lost brain tissue. Neural stem cells (NSCs) have drawn much attention because of their therapeutic potential for neurological disorders and because of their ability to differentiate into functional neuronal cell types [3]–[6]. Since the adult mammalian central nervous system (CNS) is limited in its capacity to utilize endogenous NSCs to repair neurologic deficits, cell replacement therapy can offer a potential means to recovery from the disability associated with neuronal loss. Much evidence suggests that transplanted NSCs can play a vital role in functional recovery in various animal models of CNS disorders including Parkinson's disease, Huntington's disease, stroke, and spinal cord injury [7]–[15]. In particular, NSC transplantation has recently been shown to restore brain function in animal models of TBI [16], [17]. Despite intensive research, the severe conditions (oxidative stress, necrosis, inflammation) at the site of the injury are not favorable for the survival of grafted stem cells, thus limiting the effectiveness of stem cell therapy. To overcome this problem, a variety of methods for the introduction of neural stem cells that secrete growth factors, such as brain-derived neurotrophic factor (BDNF), have been investigated for the improvement of motor function in TBI models [18].

Gel- or solid-type biocompatible scaffolds have proven invaluable for therapy aimed at reconstitution of the injured brain tissue, since they not only provide the grafted stem cells with structural support and a three-dimensional (3D) environment for improved cell adhesion and proliferation, but also can directly induce stem cell differentiation in 3D cultures [19]–[23]. Commercially available scaffolds composed of extracellular matrix have been utilized for research and clinical purposes [24]. In this study, we used an electrospun-nanofibrous poly-l-lactic acid (PLLA) polymer scaffold. This biomaterial has proven to be biodegradable, biocompatible, and non-toxic, and is FDA-approved. Our previous research regarding PLLA scaffolds was conducted in the subcutaneously engrafted mouse model of cell/scaffold complexes, and the survival duration of the grafted stem cells was monitored *in vivo* [25].

Previously, a remarkable study examined extensively the *in vivo* behavior of polyglycolic acid (PGA)-encapsulated implanted neural stem cells and found effects such as enhanced NSC differentiation and reciprocal interactions with host cells in the injured brain [26]. This study aimed to provide fluorescence-based microscopic information to evaluate the *in vivo* characteristics of implanted neural stem cells within scaffold in an invasive manner, with the need for animal sacrifice. Therefore, the non-invasive monitoring system to be able to evaluate the supportive effect of biocompatible scaffold for viable grafted stem cells is required in brain injured condition.

For noninvasive monitoring, various imaging modalities, including positron emission tomography (PET), single-photon emission computed tomography (SPECT), magnetic resonance imaging (MRI), and bioluminescence imaging, are commonly applied to living animal models. In particular, bioluminescence imaging has been widely used for noninvasive and highly sensitive visualization of implanted stem cell localization, proliferation, and migration. Bioluminescence imaging based on the light-emitting firefly luciferase reporter gene continues to be popular because it is simple, cost-effective, and uses hypersensitive instrumentation especially free from background auto-luminescence. The luminescence observed is the light produced when luciferase catalyzes the conversion of d-luciferin to oxy-luciferin, in the presence of ATP and O_2_ in living cells [27], [28].

In this study, we used a corticectomized rat, with ablation of the motor cortex as a proper brain injury model [29]. Ablation models of the motor cortex have been applied to investigate brain plasticity and drug treatments for motor deficits induced by motor cortex injury [30]. The symptoms of the motor-cortex-resected corticectomy includes decreased consciousness, limb weakness, paralysis, seizures, and involuntary movement [29], [31], [32], indicating that the motor cortex is important for muscular and behavioral control. Physically, motor-cortex-ablated rats can be considered an ideal TBI model, since it allows easy implantation of solid-type scaffolds into the damaged brain cavity, and it provides a clearly abnormal behavioral pattern with minimal variation across individual animals.

In the present study, through *in vivo* bioluminescence imaging in motor-cortex-ablated rats, we investigated *in vivo* survival of human neural stem cells dependent on the mechanical support provided by biocompatible PLLA scaffolds.

## Materials and Methods

### Cell culture

HB1.F3 human neural stem cells were initially isolated from embryonic brains at 15 weeks gestation and immortalized by retroviral transduction with the v-myc oncogene. The F3 cells were maintained in Dulbecco's modified Eagle's medium (Invitrogen, Grand Island, NY) with l-glucose and l-glutamine, containing 10% (v/v) fetal bovine serum (Invitrogen) with 10 U/mL penicillin and 10 µg/mL streptomycin (Invitrogen) in a humidified incubator at 37°C and 5% CO_2_. The cultures were passaged every 3 days by treating them with 3 mL 0.25% trypsin (w/v) and 1 mM EDTA (Invitrogen) per T75 flask for 1 min at 37°C. They were then harvested with the culture medium, centrifuged, and then resuspended in fresh flasks (Thermo Fischer Scientific, Roskilde, Denmark). The cell stock was supplemented with 10% DMSO, stored at −80°C in a freezer, and then transferred to liquid nitrogen.

### Preparation of PLLA scaffold

The fibrous scaffolds were fabricated by the wet spinning method. The PLLA solution (6%) was prepared in methylene chloride/acetone (9∶1 v/v). The polymer solution was loaded into a syringe, which was placed in a syringe pump. A blunt-tipped needle (27G) was used and the tip of the needle was immersed in a coagulation bath filled with methanol. The flow rate was between 0.9 and 1.1 mL/h and the polymer fiber was immediately formed in the bath. The collected PLLA fibers were freeze-dried to eliminate the organic solvent. Prior to cell seeding, PLLA-based scaffolds were cleaned with 70% isopropyl alcohol overnight, and were washed using phosphate buffered saline (PBS) three times. To help cell attachment to the microfibers of the PLLA scaffold, the cell-seeded scaffold was incubated for 2 h in a CO_2_ incubator and complete medium was carefully added.

### Luciferase reporter gene and retroviral modifications

To visualize the transplanted neural stem cells, F3 cells were engineered using retroviruses to express the enhanced firefly luciferase gene (*effLuc*) modified by codon optimization [33]. The DNA backbone in retroviral vector contained a Thy1.1 (CD90.1) marker and the *effLuc* gene linked to an internal ribosome entry site (IRES), under the control of the cytomegalovirus (CMV) promoter. To produce retroviruses, an *effLuc* viral vector and a DNA vector carrying major structural proteins (GAG, Pol, and Env) were transfected into a 293FT packaging cell line seeded in a 10-cm flask dish and 48 h after transfection, the supernatant containing retroviruses was collected. The produced retrovirus was added to F3-effLuc cells with 10 mM polybrene. The infected cells were separated into CD90.1^+^ and CD90.1^−^ by magnetic-activated cell sorting (MACS; Miltenyi Biotech Ltd., Bisley, Surrey, UK) using monoclonal anti-CD90.1 conjugated to magnetic microbeads. The purity of CD90.1^+^ cells were identified by FACS (BD Immunocytometry System; Becton Dickinson, CA, USA) analysis using the monoclonal antibody, anti-CD90.1, conjugated to fluorescein isothiocyanate (FITC).

### Flow cytometry

We incubated 1×10^6^ F3 or F3-effLuc cells in FACS buffer (PBS with 5% fetal bovine serum and 0.05% sodium azide) for 30 min at 4°C. The cells were fixed using 2% paraformaldehyde fixation buffer in PBS, and suspended cells in 100 µL of permeabilization buffer (0.1% Triton X-100 in PBS), followed by incubation at room temperature for 30 min. F3 and F3-effLuc cells were stained with antibodies (Becton Dickinson) for the human proteins, CD44, Nestin, Sox1, Sox2, GFAP, and Ki-67 in FACS buffer for 30 min. After staining, each cell-type was resuspended in FACS buffer and analyzed using a FACSCalibur system with CellQuest software (Becton Dickinson).

### Scanning electron microscopy

The morphology of F3-effLuc cells seeded into PLLA scaffolds were characterized with field emission scanning electron microscopy (SEM; JEM-7401F, Joel Ltd., Tokyo, Japan) on 1 and 4 days. The samples were fixed using 1 mL of 2% glutaraldehyde (Sigma-Aldrich, MO, USA) at 4°C for 2 hr. To prepare dried and hydrated samples, they were washed with PBS and dehydrated by soaking in increasing concentrations of ethanol (30–100%). Specimens were covered with gold-palladium alloy on an aluminum stub after drying in hexamethyldisilazane (HMDS).

### Corticectomy and the animal model

Animal were maintained without unnecessary pain or distress, and all animal experiments were approved by Seoul National University Hospital Institutional Animal Care and Use Committee (IACUC NO. 13-2010-005-0). Six-week-old adult male Sprague-Dawley (SD) albino rats weighing 180–200 g were provided by the Clinical Research Institute of Seoul National University Hospital. Rats were anesthetized with zoletil 50 (75 mg/kg, i.p.) and xylazine (10 mg/kg, i.p.). The anesthetized rats were placed in a stereotactic frame, and the height was adjusted between lambda and bregma. The midline scalp and temporal muscles were incised, and the exposed skull was stereotactically removed using a hand drill at the following coordinates: A) 4 mm rostral and 1 mm lateral, B) 2 mm caudal and 1 mm lateral, and C) 4 mm rostral and 6 mm lateral. After removing the skull, the exposed dura and motor cortices were ablated with a surgical blade, and the resected area was filled with Gelfoam (Pharmacia and Upjohn, Kalamazoo, MI, USA). Finally, the incised skin was sutured.

### Limb placement behavior test in corticectomized rats

Two types of proprioceptive-response-related behavior tests were conducted to evaluate the corticectomized rat model: the whisker tactile and forelimb tests. Behavioral tests were carried out in semi-darkness in a silent room to minimize the impact of other environmental stimulation. The proprioceptive forelimb observation test was performed on an experimental table by gently pulling down the forelimb of corticectomized rats to examine the degree of forelimb retraction. Scores for the test were calculated as follows: 0 for normal retraction and 1–3 for abnormal retraction according to the amount of stretching. The whisker tactile test evaluated the level of sensory perception of whisker stimulation. The test examined whether the rat's forelimb reached out to the table when its whiskers approached within 2 mm of the table surface. Each animal was tested three times, and the scores were calculated as follows: 0 for normal reaching, and 1–3 for abnormal reaching according to the amount of stretching.

### In vitro luciferase assay and in vivo bioluminescence imaging

*In vitro* luciferase assays were performed using luciferase assay kits (Applied Biosystems, Carlsbad, CA, USA). F3-effLuc cells were seeded onto 24- or 6-well plate and 24 h after plating, the cells were washed using PBS, and lysed using lysis buffer. The lysated F3-effLuc cells were transferred to a 96-well plate for detection of the bioluminescence signal. Luciferase intensity was measured using a Varioskan Flash (Thermo Fisher Scientific, Vantaa, Finland) at an acquisition time of 1 s. For *in vivo* imaging, the wet-spinning PLLA scaffold was pre-wetted overnight using 70% v/v isopropyl alcohol, and washed three times with PBS. For implantation of cell/scaffold complex, 2×10^6^ F3-effLuc cells were harvested using trypsin, and resuspended in PBS. Then, 40 µL of resuspened F3-effLuc cells were incorporated into the PLLA scaffold, and after the F3-effLuc/PLLA complex was incubated for 2 h, it was implanted into the cavity of the corticectomized rat brain (n = 3). For acquisition of the bioluminescence images, the rats were sedated with 2% isoflurane in 100% O_2_ through a nose cone. d-Luciferin (Caliper Life Sciences, Hopkinton, MA, USA) was diluted to 3 mg/100 µL in normal saline and 0.6 mg of d-Luciferin was directly administrated into the brain on 0, 1, 3, 5, 8, 11, and 14 days post-transplantation. To suppress the innate immune response against the human F3-effLuc cells, cyclosporine A (5 mg/kg) was intraperitoneally administered every day after transplantation. An IVIS-100 imaging system equipped with a CCD camera (Caliper Life Sciences) was used for *in vivo* bioluminescence imaging. Images were acquired by integrating light for 5 min. The luminescence intensity in regions of interest from each image was quantified to examine the viability of the implanted cells.

### Brain sectioning and histological analysis

Rats were anesthetized and transcardially perfused with normal saline containing heparin and 4% paraformaldehyde (PFA; Sigma-Aldrich). The rats' brains were removed, post-fixed in 4% paraformaldehyde for 24 h, and dehydrated in 10%, 20%, and 30% sucrose at 4°C. Specimens were frozen in OCT medium (Leica, IL, USA) and then, 14-µm-thick coronal serial sections were cut and mounted on gelatin-coated slides. One brain section in each group was processed with basic hematoxylin and eosin (H&E) staining, and six sets of sections were immunohistochemically stained. Sections on the glass slides were permeabilized in PBS containing 0.5% Triton-X for 5 min at 4°C, rinsed 3 times with PBS for 5 min, and then incubated in 1% normal horse serum for 1 h at room temperature. The slides were incubated overnight in a 1∶500-diluted solution of anti-TuJ1 (Millipore Co., Billerica, MA, USA), anti-luciferase (Millipore Co.). Localization of transplanted NSCs was investigated via staining with anti-Thy1.1 and anti-luciferase antibodies. Immunohistochemistry analyses were performed using confocal laser microscopy (LSM 510; Carl Zeiss, Jena, Germany).

### Statistical analysis

Data are expressed as means ± standard errors of means (SEM) from six biological replicates and were calculated using the Student's *t*-test. Statistical significance was accepted at P value of <0.005.

## Results

### Establishment of F3 cells stably expressing the effLuc transgene

**Figure 1:**
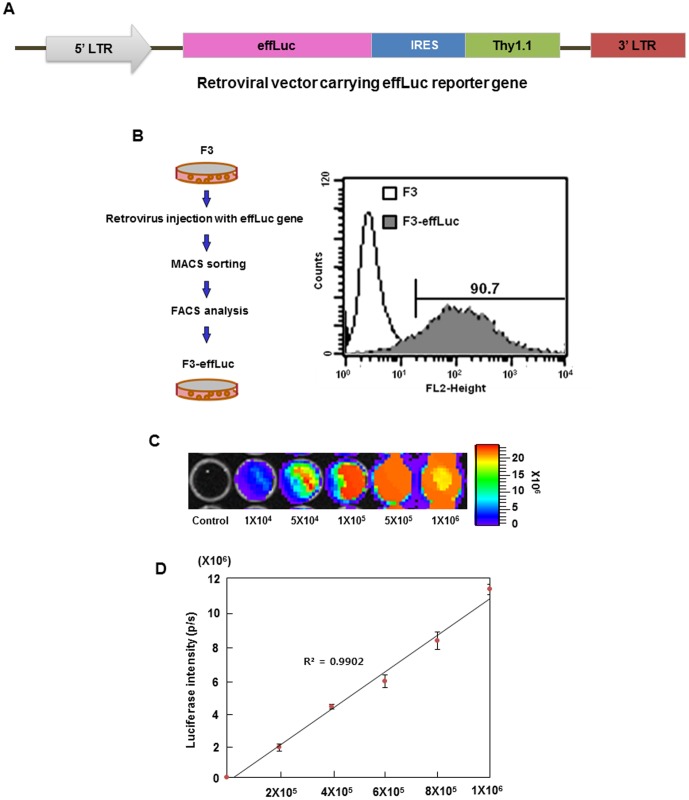
*In vitro* luciferase reporter activity in F3-effLuc cells. (A) Retroviral construct that contains the *effLuc* gene and Thy1.1 (CD90.1), linked with an IRES (internal ribosomal entry site). (B) Magnetic-activated cell sorting (MACS) was performed to collect the F3-effLuc cells. Flow activated cell sorting (FACS) analysis showed that more than 90% of cells were successively transfected with the *effLuc* vector. (C) The luciferase activity (n = 3) of F3-effLuc cells cultured in a 96-well plate were measured using an IVIS-100 optical imaging device. Firefly luciferase activity continuously increased in F3-effLuc cells in proportion to cell number, and (D) quantitative analysis showed a linear relationship between the cell number and bioluminescence signals.

To establish genetically engineered F3 human neural stem cells for visualizing *in vivo* characteristics of the implanted stem cells, we used F3 neural stem cells stably expressing the codon-optimized enhanced firefly luciferase gene and a Thy1.1 (CD90.1) marker linked with IRES under the control of the CMV promoter in the retroviral DNA backbone (Fig. 1A). CD90.1^+^ F3-effLuc cells in a heterogeneous cell population were collected via MACS. To measure the transduction efficiency of the infected cells, FACS analysis was conducted on the collected cells. The results indicated that these cells were 90.7% F3-effLuc (Fig. 1B). The luciferase intensity in the 96-well microplate showed the gradually increasing pattern as the number of F3-effLuc increased (Fig. 1C). The luciferase activity and F3-effLuc cell number were significantly correlated via a quantitative luminometric assay (Fig. 1D).

### Examination of the characteristics of stem cells transfected with effLuc

**Figure 2:**
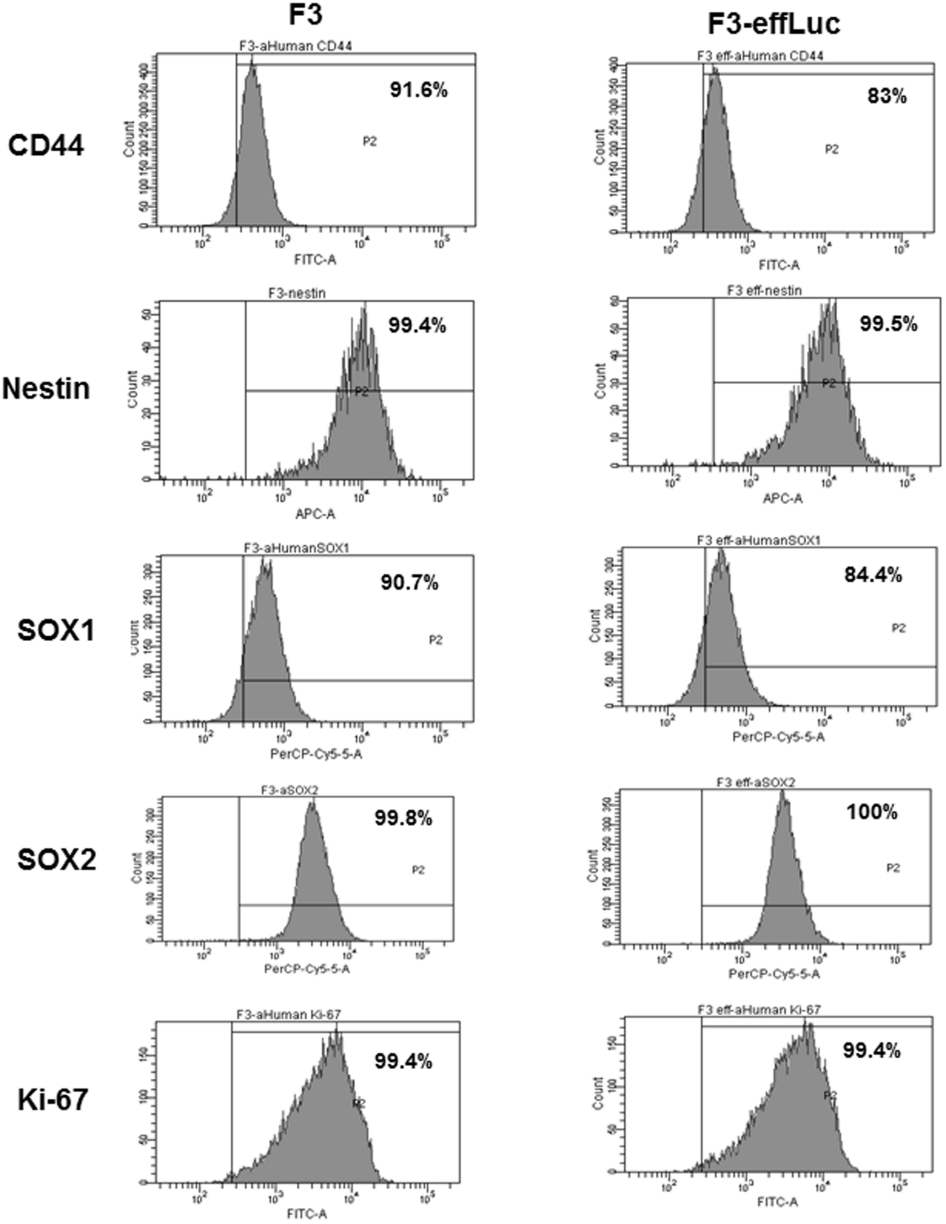
Validation of stem cell characteristics in F3 cells and F3-effLuc cells. Flow cytometric analysis showed F3 and F3-effLuc cells are positive for the stem cell surface marker, (a) CD44, and the intracellular marker s, (b) Nestin, (c) Ki67, (d) Sox1, and (e) Sox2.

To test whether the *effLuc* transgene influenced F3 stem cell characteristics, four different proteins involved in stem cell self-renewal were examined in non-transfected F3 cells and F3-EffLuc cells. FACS analysis showed that more than 90% of F3 cells were positive for the four stem cell markers (CD44, Nestin, SOX1, and SOX2). Expression levels of all four markers were similarly high (almost identical) in transfected cells, indicating that the transgene did not affect the stem cell properties (Fig. 2). No difference in Ki-67 (proliferation marker) expression was observed between F3 and F3-effLuc cells.

### Spatial distribution and proliferation of F3-effLuc cells within the PLLA scaffold

**Figure 3:**
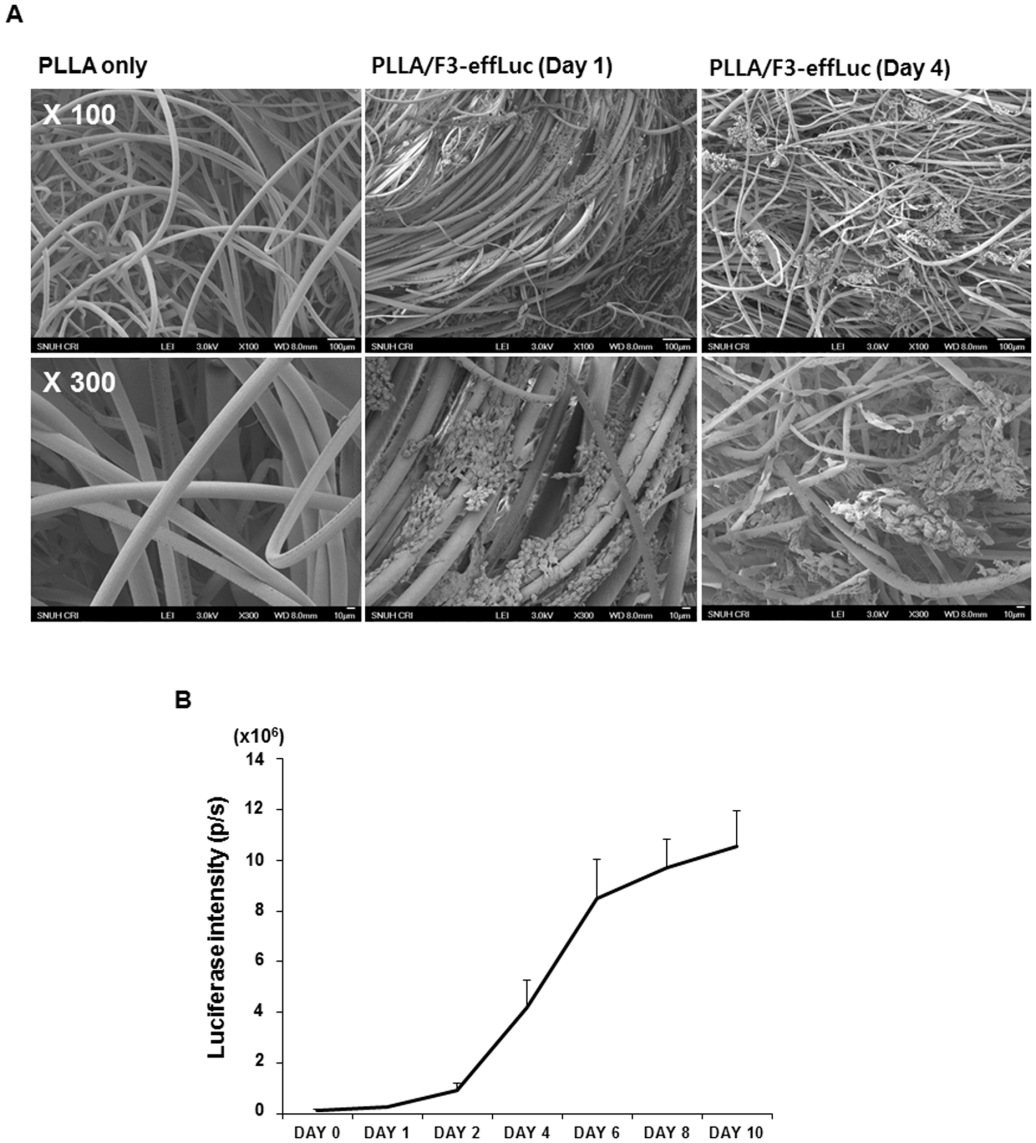
*In vitro* proliferative effect in F3-effLuc cells within the PLLA scaffold. (A) Scanning electron microscope (SEM) analysis was conducted to confirm adhesion of F3-effLuc cells to the PLLA scaffold. SEM images showed that F3-effLuc cells were stably attached onto the microfibers of the PLLA scaffold. (B) The luciferase intensity was quantified after F3-effLuc cells were incubated with the sterile PLLA scaffold. F3-effLuc cells incorporated within the PLLA scaffold were stably proliferated at 10 days.

To examine whether genetically modified F3-effLuc cells were stably attached to the synthetic PLLA microfiber scaffold, we examined the reciprocal interaction of F3-effLuc with the pre-wet PLLA scaffold. F3-effLuc cells (5×10^5^) were incorporated within a sterile PLLA scaffold, and incubated for 2 h to allow the F3-effLuc cells to evenly distribute throughout the scaffold. The porous microstructure of the wetspun microcomposite PLLA scaffold (pore size distribution: 50–200 µm) was clearly observed under SEM [34]. F3-effLuc cells grown in PLLA scaffolds robustly adhered, and showed widespread cell attachment within the whole PLLA scaffold (Fig. 3A). To examine whether the engineered F3-effLuc cells proliferated well within the microfiber PLLA scaffolds, F3-effLuc cells were seeded into sterile PLLA scaffolds and incubated until 10 days in 24 well plate. An *In vitro* luciferase assay showed that F3-effLuc cell proliferation inside the PLLA scaffold gradually increased up to 2 days. Exponential proliferation was found to occur from 2 days (Fig. 3B).

### Evaluation of the corticectomy rat model through behavioral testing

**Figure 4:**
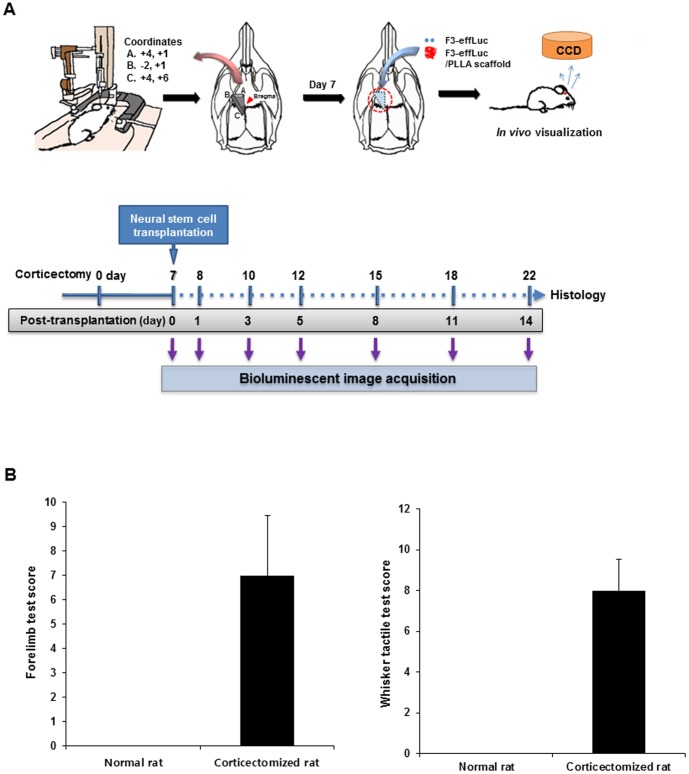
Schematic representation of the procedure for *in vivo* optical imaging. (A) The protocol is for *in vivo* monitoring of F3-effLuc cells implanted in a corticectomized rat model. The motor cortex region of the Sprague-Dawley rat brain was surgically removed at the given coordinates and after 7 days, the rats were transplanted with F3-effLuc cells alone or F3-effLuc/PLLA scaffold complexes, and then administered cyclosporine A everyday. The grafted cells were monitored at 0, 1, 3, 5, 8, 11, and 14 days using a bioluminescence-imaging device. At the end of the implant period, histological analyses were performed using hematoxylin and eosin (H&E) staining and immunohistochemistry. (B) Behavior tests were performed 7 days after motor cortex ablation. The traumatic brain injury (TBI) models were evaluated by forelimb placing tests and whisker tactile tests in normal and corticectomized rats (n = 10).

The motor cortex region in the rat brain was stereotactically removed at three coordinates from bregma (Fig. S1), and then F3-effLuc or F3-effLuc/PLLA cells were implanted in the corticectomized rat brain at 7 days after the operation. The complete experimental procedure is summarized in Fig. 4A. The implanted F3-effLuc cells were monitored at 0, 1, 3, 5, 8, 11, and 14 days using local administration of d-luciferin and a bioluminescence imaging device. The behavior tests indicated that the corticectomized rats showed abnormal behavior compared to control rats, exhibiting limb weakness and no response to whisker stimuli (Fig. 4B). The motor-cortex-ablated rats clearly displayed impaired motor function, scoring highly for abnormality.

### In vivo bioluminescence imaging of PLLA-encapsulated transplanted F3-effLuc cells in the corticectomized rat model

**Figure 5:**
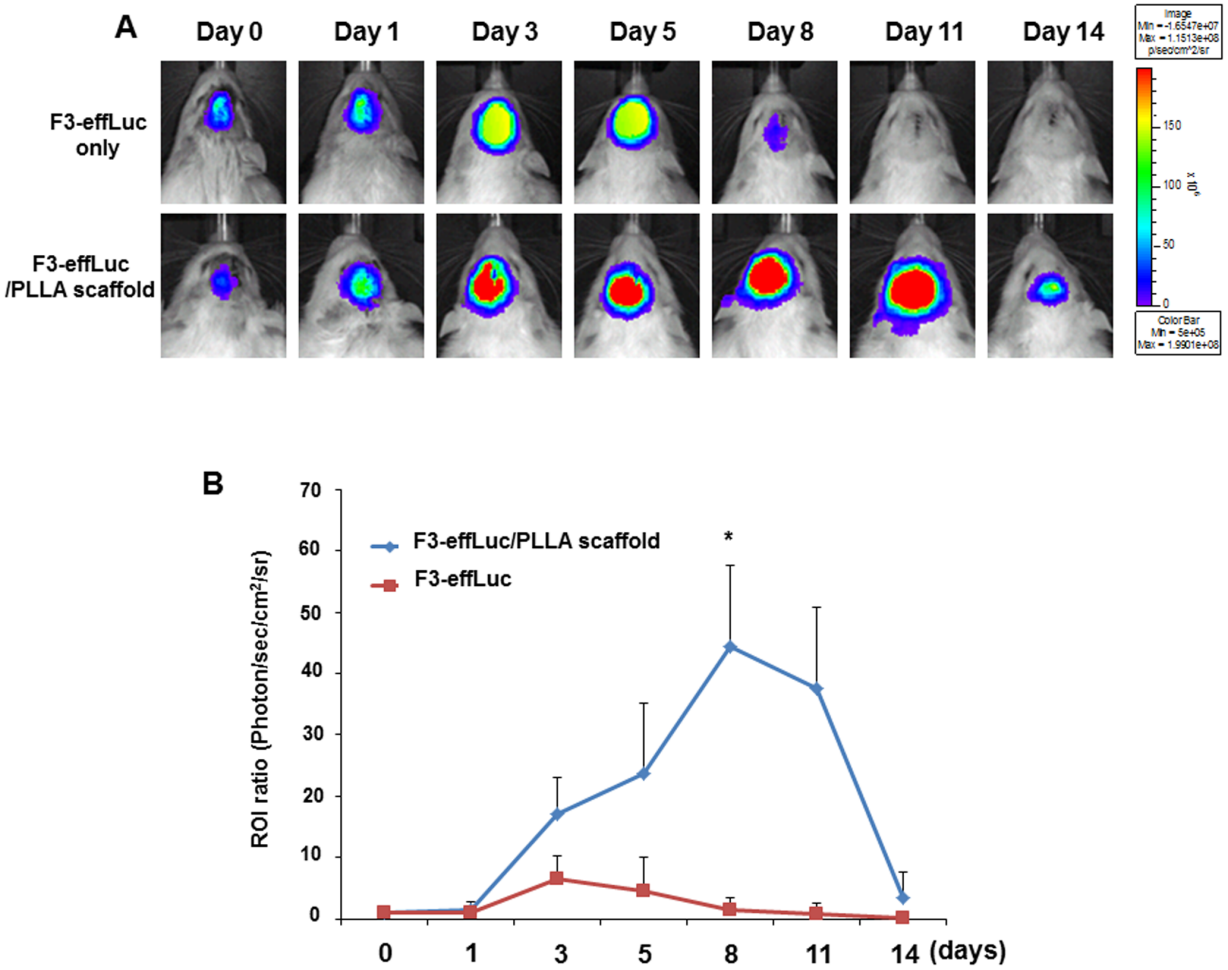
*In vivo* bioluminescence imaging of the implanted F3-effLuc/PLLA scaffold in a corticectomized rat model. (A) After F3-effLuc cells were incubated within the PLLA scaffold for 2 hr, the cell/scaffold complex was implanted into the ablated motor cortex area of the rat brain. Firefly luciferase bioluminescence imaging was performed over 14 days. The prolonged luminescence signals in F3-effLuc cells within the PLLA scaffold were clearly visualized in the ablated area. (B) Quantitative ROI analysis showed significantly enhanced survival duration for F3-effLuc cells within the PLLA scaffold (n = 6). P value, * <0.005.

On post-operative 7 days, 1×10^6^ F3-effLuc cells were harvested for seeding of the PLLA scaffold. After the harvested F3-effLuc cells were incubated for 2 h with the PLLA scaffold to keep F3-effLuc cells well attached within the scaffold, F3-effLuc cells were implanted into the ablated area with or without a PLLA scaffold. d-Luciferin was locally injected into the brain, and bioluminescence images were acquired up to 14 days. Immediately after the cells were implanted, bioluminescence signals were clearly observed in both the F3-effLuc-only group and the F3-effLuc/PLLA group. Gradually increasing signals were detected up to 5 days in both groups, showing a significantly increased luciferase signal 3 days after transplantation. The luciferase activity in the cell-only group was decreased dramatically at 8 days and eventually disappeared at 11 days (Fig. 5A, n = 3). However, in the F3-effLuc/PLLA group, intense bioluminescence activity was detected in the implanted area at 8 days, and this was maintained up to 14 days (Fig. 5A). These results demonstrate that the supportive effect of the PLLA scaffold increases survival duration of implanted stem cells in the corticectomized rat model. For the quantitative analysis, photon counts in the region of interest (ROI) were measured from *in vivo* bioluminescence data. *In vivo* bioluminescence images from the corticectomized rat model revealed a continuously increasing luminescence signal of longer duration for the scaffold-encapsulated cells (Fig. 5B).

### Histological analysis of PLLA-encapsulated F3-effLuc cells in the corticectomized rat

**Figure 6:**
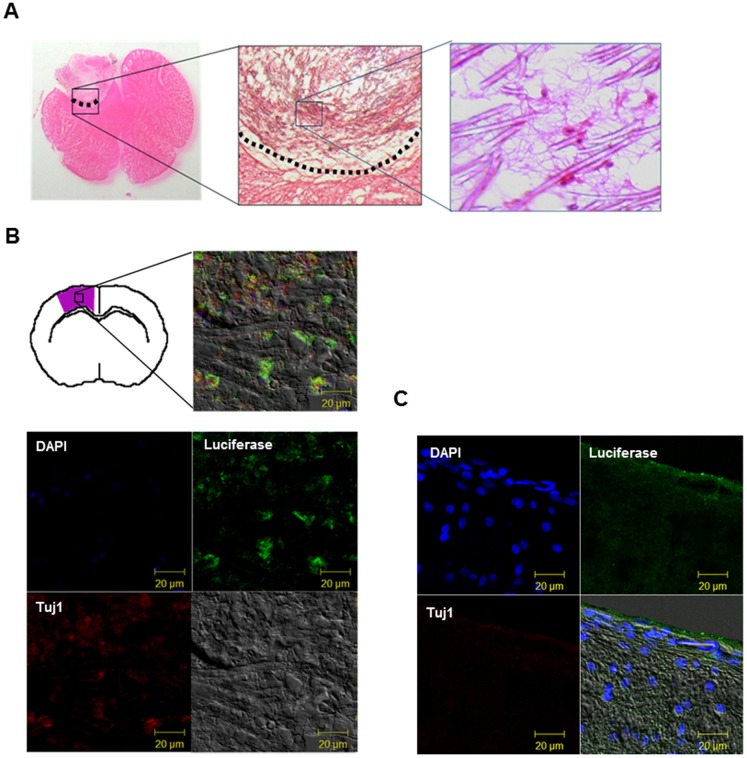
Immunohistochemistry results for PLLA-encapsulated F3-effLuc cells implanted in a corticectomized rat brain. (A) Hematoxylin and eosin (H&E) staining was performed on the corticectomized rat brains transplanted with the F3-effLuc/PLLA scaffold complex. Slices of the fixed brain were stained with hematoxylin and eosin to examine the presence of transplanted cells on the PLLA microfibers. (B) Confocal fluorescence images of the transplanted region (purple) revealed luciferase expression (green) in F3-effLuc cells was partially co-localized with the expression of Tuj1, a neuron-specific marker (red). Nuclei were visualized with DAPI (blue). Scale bars represent 20 µm. No luciferase expression was observed in F3 cell only-transplanted rat model.

For the histological analyses, the whole brain was extracted from corticectomized rats (n = 3 per group) immediately after formalin perfusion via transcardial injection. The fixed brain tissue showed that the ablated area was filled with the F3-effLuc/PLLA scaffold (Fig. S2). To evaluate the characteristics of the transplanted F3-effLuc/PLLA complex, 14-µm-thick brain slices were stained with several antibodies including those for luciferase and the early neuronal marker beta III tubulin (TuJ1). H&E staining allowed us to observe the F3-effLuc cell morphology on the fibers of the PLLA scaffold in the implanted region (Fig. 6A). To examine the differentiation pattern of transplanted F3-effLuc cells within the PLLA scaffold, brain slices were stained with a TuJ1-specific antibody. Tuj1 and luciferase expression in the F3-effLuc/PLLA slice were partially co-localized (Fig. 6B). Luciferase expression in F3 cell only-implanted rat brain was not detected at 14 days after cell implantation. This result indicates that the F3-effLuc cells within the PLLA scaffold were differentiated into the neuronal lineage about 2 weeks after implantation.

## Discussion

Newly developed scaffolds that are biochemically and physicochemically suitable for clinical use have been explored for applications associated with cell-based therapies; these include synthetic polymers such as polylactic acid (PLA) [35], polyglycolic acid (PGA) [36], and poly(lactic-co-glycolic acid) (PLGA) [37], as well as natural biomaterials such as hydrogel [38], gelatin [39], chitosan, and collagen [40], [41].

Numerous studies have investigated the use of stem cells for cell replacement therapy because stem cells have the potential to replace tissue deficits that occur in brain injury via their direct implantation into the damaged area. NSCs are especially suitable for implantation treatments of neurodegenerative diseases, and they have been widely used in neurological disease models. To improve the survival duration of implanted stem cells, biocompatible scaffolds have been explored for use in tissue engineering. PLLA scaffold functions as a bioartificial niche, providing a 3D structure that enhances cell survival and proliferation *in vivo*. We have previously reported that the survival of implanted stem cells within scaffolds can be compared to that of scaffold-free cells through optical or radionuclide imaging [25]. In this study, we applied this image-based methodology to the brain injury model, demonstrating enhanced survival and proliferation rates for scaffold-encapsulated transplanted NSCs.

Our results confirmed the supportive effects of PLLA scaffolds for implanted stem cells in the corticectomized rat model, by evaluating the *in vivo* viability of transplanted stem cells using bioluminescence imaging. When F3-effLuc/PLLA scaffold complexes were transplanted into the ablated motor cortex, survival of transplanted stem cells was maintained for a longer time, and the proliferation rate was greater in the presence of the scaffold (Fig. 5A). Directly after F3-effLuc cell implantation, the proliferation rate of these cells increased gradually up to 5 days without the scaffold, and up to 8 days with the scaffold. A dramatic decline in cell survival occurred after 5 days without the scaffold, and after 11 days with the scaffold (despite daily immunosuppressant treatment). We suggest that the decline in survival was because of the environment around the cortical lesion, where implanted cells would be subjected to immunological and necrotic insults from the host cells. Despite the harsh environment, the survival duration of PLLA-encapsulated implanted F3-effLuc cells was significantly longer on account of the protection given by the fibrous scaffold.

Co-immunostaining results showed that the expression of the early neuronal marker Tuj1 was detected in the F3-effLuc/PLLA cells about 2 weeks after implantation in corticectomized region, which demonstrated that the cells within the PLLA scaffold had entered the neuronal lineage. This result indicates the possibility of improving functional recovery of brain tissue via enhanced neuronal differentiation resulting from the presence of the PLLA scaffold.

In the present study, we used a noninvasive reporter gene system to show that human NSCs transplanted into the ablated region exhibited increased viability thanks to the protection provided by a PLLA scaffold. We also confirmed that scaffold-encapsulated stem cells implanted into the injured sites began to be well differentiated along the neuronal lineage and had higher survival rates. It is important to note that long-term survival of transplanted cells is a fundamental requirement for their use in improving recovery in animal models of brain injury. To further enhance survival duration, surface-engineered PLLA scaffolds coated with tropic factors such as fibroblast growth factor (FGF) could be used [34].

In the current study, we showed that implanted human NSCs incorporated within PLLA scaffolds were clearly visualized in motor-cortex-ablated rats. In particular, the supportive effect of a biocompatible PLLA scaffold, playing the role of a bio-bridge *in vivo,* was proven by using a noninvasive luminescence imaging technique in a traumatic injury model. Thus, the survival of stem cells implanted in a cortex-resected animal can be prolonged by using a polymeric scaffold to support implanted cells *in vivo*. *In vivo* imaging techniques for stem cell implantation in models of brain disorders are useful because they allow easy monitoring of implanted-cell survival. Scaffold-mediated implantation can play a supportive role in the treatment of trauma injury by enhancing implanted cell survival. Therefore, this study could provide useful insights to follow up stem-cell-based therapeutic research and for clinical applications.

## Supporting Information

Figure S1 **The corticectomized rat model. Surgical resection of the motor cortex was carried out at three coordinates (red circles).** * bregma region.
(TIF)

Figure S2 **Comparison of corticectomized rat brains bearing the (a) F3-effLuc cells and (b) F3-effLuc/PLLA scaffold complex.**
(TIF)
