# Supplementary figures and images for: In Vivo Bioluminescence Imaging for Prolonged Survival of Transplanted Human Neural Stem Cells Using 3D Biocompatible Scaffold in Corticectomized Rat Model
Source: PLoS One. 2014 Sep 8;9(9):e105129. doi: 10.1371/journal.pone.0105129 (PMC4157740; doi:10.1371/journal.pone.0105129)

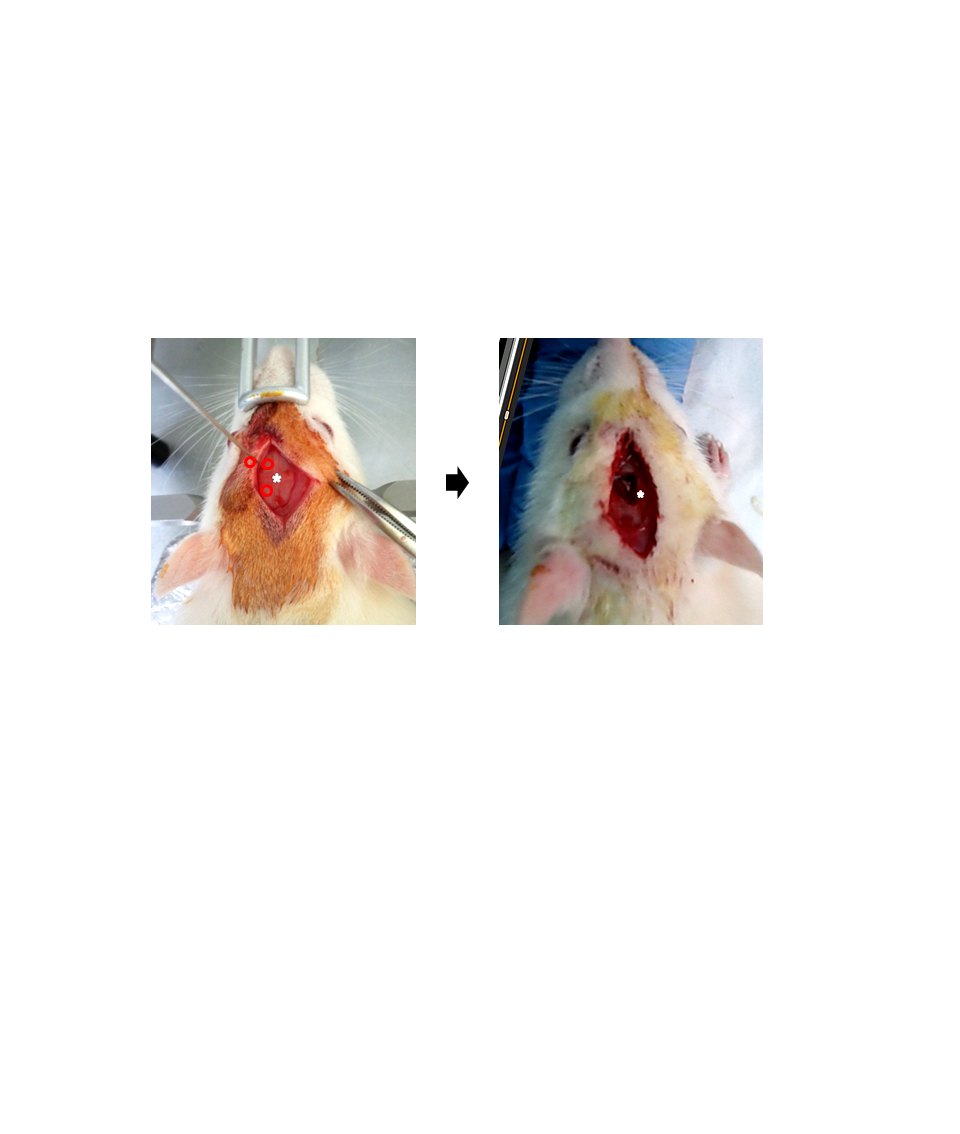

Supplement: Figure S1 — The corticectomized rat model. Surgical resection of the motor cortex was carried out at three coordinates (red circles). * bregma region. (TIF) [file pone.0105129.s001.tif]

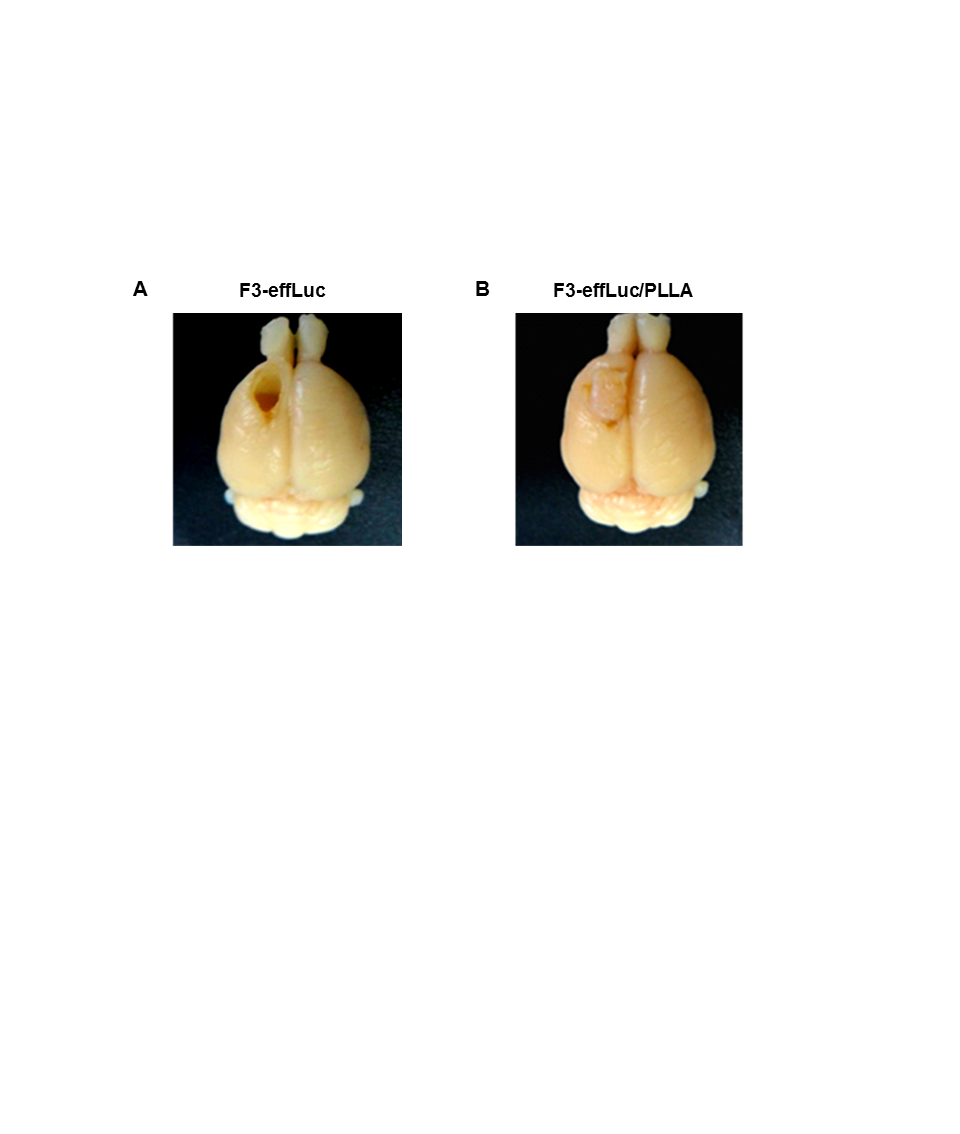

Supplement: Figure S2 — Comparison of corticectomized rat brains bearing the (a) F3-effLuc cells and (b) F3-effLuc/PLLA scaffold complex. (TIF) [file pone.0105129.s002.tif]
